# Application of whole genome sequencing to query a potential outbreak of *Elizabethkingia anophelis* in Ontario, Canada

**DOI:** 10.1099/acmi.0.000017

**Published:** 2019-04-24

**Authors:** Lisa R. McTaggart, Patrick J. Stapleton, AliReza Eshaghi, Deirdre Soares, Sylvain Brisse, Samir N. Patel, Julianne V. Kus

**Affiliations:** 1 Public Health Ontario, 661 University Avenue, Toronto, ON, Canada M5G 1M1; 2 Department of Laboratory Medicine and Pathobiology, University of Toronto, Toronto, ON, Canada; 3 Institut Pasteur, Biodiversity and Epidemiology of Bacterial Pathogens, F-75724 Paris, France

**Keywords:** *Elizabethkingia anophelis*, outbreak, whole genome sequence, core genome MLST, antimicrobial resistance

## Abstract

Bioinformatic analysis of whole genome sequence (WGS) data is emerging as a tool to provide powerful insights for clinical microbiology. We used WGS data to investigate the genetic diversity of clinical isolates of the bacterial pathogen *Elizabethkingia anophelis* to query the existence of a single-strain outbreak in Ontario, Canada. The Public Health Ontario Laboratory (PHOL) provides reference identification of clinical isolates of bacteria for Ontario and prior to 2016 had not identified *E. anophelis *. In the wake of the Wisconsin outbreak of 2015–2016 for which a source was never elucidated, the identification of *E. anophelis* from clinical specimens from five Ontario patients gave reason to question the presence of an outbreak. Genomic comparisons based on core genome multi-locus sequence typing conclusively refuted the existence of an outbreak, since the 5 Ontario isolates were genetically dissimilar, representing at least 3 distinct sub-lineages scattered among a set of 39 previously characterized isolates. Further interrogation of the genomic data revealed multiple antimicrobial resistance genes. Retrospective reidentification via *rpoB* sequence analysis of 22 clinical isolates of *Elizabethkingia* spp. collected by PHOL from 2010 to 2018 demonstrated that *E. anophelis* was isolated from clinical specimens as early as 2010. The uptick in *E. anophelis* in Ontario was not due to an outbreak or increased incidence of the pathogen, but rather enhanced laboratory identification techniques and improved sequence databases. This study demonstrates the usefulness of WGS analysis as a public health tool to quickly rule out the existence of clonally related case clusters of bacterial pathogens indicative of single-strain outbreaks.

## Introduction

*Elizabethkingia* species are aerobic, non-motile, Gram-negative bacilli that are ubiquitous in soil and freshwater [1]. Although characterized as environmental bacteria, they are occasionally isolated from hospital environments and clinical specimens. They do not normally inhabit the human body. Opportunistic infections are rare but problematic, as *Elizabethkingia* spp. are naturally resistant to a wide range of antimicrobial agents [1]. Following the original description of the type species *Elizabethkingia meningoseptica* (previously named *Flavobacterium meningosepticum* and *Chryseobacterium meningosepticum *) as the cause of a case of neonatal meningitis in 1959 [2], the genus has expanded to contain six species, including *Elizabethkingia miricola *, *Elizabethkingia anophelis* (including strains previously described as *Elizabethkingia endophytica *), ‘* Elizabethkingia bruuniana *’, ‘* Elizabethkingia ursingii *’ and ‘* Elizabethkingia occulta *’ [3]. (The latter three names have no standing in the nomenclature.) Due to the increased incidence of *Elizabethkingia* bacteraemia over the past decade, *E. meningoseptica* and *E. anophelis* in particular are considered to be emerging pathogens [4, 5].

In 2015–2016, an outbreak involving 66 laboratory-confirmed infections of *E. anophelis* occurred in the US states of Wisconsin, Illinois and Michigan [6]. By far the largest documented *Elizabethkingia* outbreak, it was also unique in that most cases manifested in community settings, unlike previous healthcare-associated outbreaks [7–9]. Despite extensive investigation, the source of the infection was never identified and the outbreak spontaneously resolved by the end of 2016 [6]. Whole-genome sequence (WGS) analysis was instrumental in confirming the outbreak by demonstrating the high degree of genetic similarity between outbreak strains, which suggested that it was caused by a single strain from a single source [6]

Prior to 2016, the Public Health Ontario Laboratory (PHOL), which is a reference laboratory for the province of Ontario (population 14.3 million), had never reported an isolate of *E. anophelis *, but between July 2016 and February 2018, five clinical isolates from patients located in the greater Toronto area were received and subsequently identified as *E. anophelis *. Given the seriousness of *Elizabethkingia* infections due to their intrinsic resistance to several antibiotics [10], the relatively close geographical proximity of Toronto, Ontario to the Wisconsin outbreak, and the uncertainly surrounding the source of the aforementioned outbreak, we sought to use WGS analysis to confirm or conclusively rule out the presence of an *Elizabethkingia* outbreak strain in Ontario. Furthermore, we performed a retrospective reidentification of all *Elizabethkingia* sp. isolates received by PHOL during the preceding years to determine whether the recent uptick in the number of *E. anophelis* clinical isolates was due to a potentially increased incidence of infection or due to the implementation of more accurate clinical laboratory bacterial identification methods and improved availability of nucleotide sequences of *E. anophelis* in public databases.

## Methods

### Initial identification

PHOL, Toronto, Ontario received five clinical isolates (blood *n*=1, urine *n*=1, aspirate *n*=1, endoscopy specimen *n*=1, fluid *n*=1) of unknown Gram-negative bacteria, obtained from five individual patients in the greater Toronto area between July 2016 and February 2018 for identification and susceptibility testing. The isolates were originally identified by Bruker matrix-assisted laser desorption/ionization time-of-flight mass spectrometry (MALDI-TOF MS) (Bruker Daltonics, Billerica, MA, USA) using the MALDI Biotyper Reference Library v5 (isolate PHOL-090), v6 (isolate PHOL-515) or v7 (isolates PHOL-537, PHOL-104, PHOL-785) (Bruker Daltonics), with each isolate yielding match scores ≥2.0 to both *E. meningoseptica* and *E. miricola *. It is our experience, and the experience of others [3, 11], that the Bruker MALDI-ToF MS cannot differentiate between *Elizabethkingia* species; it frequently returns multiple species IDs with scores ≥2.0. Therefore, in our institution, all *Elizabethkingia* isolates are reflexed to partial 16S rDNA PCR and sequence analysis using primers 8FPL 5′-AGTTTGATCCTGGCTCAG-3′ and 806R 5′-GGACTACCAGGGTATCTAAT-3′ to yield a 748 bp fragment for identification. 16S rDNA sequences were subjected to a blast search against the NCBI GenBank nucleotide database [12, 13] with the interpretation criteria described in the Clinical Laboratory Standards Institute (CLSI) document MM18-A [14] being used to identify the isolates. 16S rDNA sequence analysis determined these isolates to be *E. anophelis *. It is important to note that none of the MALDI BioTyper Reference Libraries used for the identification of these isolates contained spectra for *E. anophelis *.

### Genome sequencing and analysis

In order to definitively identify the species, isolates underwent WGS analysis. Following DNA isolation using the Qiagen DNeasy Blood and Tissue kit (Qiagen, Germantown, MD, USA), libraries were constructed using the Nextera XT DNA library preparation kit (Illumina, Inc., San Diego, CA, USA) and sequenced on a MiSeq instrument using a 2×150 paired-end protocol. At least 30× average read depth coverage was achieved for all samples. *De novo* assemblies were generated with SPAdes v 3.9.1 [15] and annotated using Prokka v 1.13 [16]. This whole-genome shotgun project has been deposited at DDBJ/ENA/GenBank under the accession numbers RSAV00000000, RSAW00000000, RSAX00000000, RSAY00000000 and RSAZ00000000. The version described in this paper is the first version, RSAV01000000, RSAW01000000, RSAX01000000, RSAY01000000 and RSAZ01000000. The assemblies were submitted to the publicly available core genome multi-locus sequence typing (cgMLST) database at the Institut Pasteur (http://bigsdb.pasteur.fr/elizabethkingia/). Core genome analysis involving 1546 genes and genomic comparison of the 5 Ontario isolates to a selection of 38 isolates of *E. anophelis* was performed using an unweighted pair group method with arithmetic mean (UPGMA) algorithm with 1000 bootstrap replicates based on allelic profiles as previously described [4, 6]. The 39 comparative isolates were chosen from among publicly available genomes, so that they would represent all previously described phylogenetic sublineages [4, 6] of *E. anophelis *.

Because of the high level of antimicrobial resistance of *Elizabethkingia *, and as we had the whole genome sequences for each isolate, antimicrobial resistance markers were investigated. Antimicrobial resistance genes involving a protein homologue mechanism of resistance were identified using three methods: (1) the hmmscan function of HMMER3 v 3.1b2 (http://hmmer.org/) [17] against Core ResFams database v 1.2 [18], a curated database of antimicrobial resistance protein families and associated profile hidden Markov models; (2) the specialty genes for antibiotic resistance identified by PATRIC [19] following RAST annotation [20]; and/or (3) the Resistance Gene Identifier (Perfect and Strict hits only) of the Comprehensive Antimicrobial Resistance Database (CARD) [21, 22]. Antimicrobial resistance gene targets involving the protein variant mechanism of resistance were identified following gene annotation by Prokka v 1.13 [16] with sequence comparison in BioNumerics v 6.6 (Applied-Maths, Austin, TX, USA).

### MALDI-ToF MS, *rpoB* sequencing and antimicrobial susceptibility testing

In order to determine whether the 5 *E. anophelis* isolates were indeed the first isolates of this species received by PHOL from Ontario patients we examined an additional 17 *Elizabethkingia* sp. isolates received by the PHOL from 2010 to 2018. These historic isolates were originally identified by biochemical assays [23] or gas chromatographic analysis of fatty acid methyl esters (FAMEs) with the Sherlock Microbial Identification System (MIDI, Inc., Newark, DE, USA) (2010–2015) or MALDI-ToF MS and partial 16S rDNA sequence analysis (2015–2018). Retrospective reidentification was performed by Bruker MALDI-ToF MS against the MALDI Biotyper Reference Library v7 (Bruker Daltonics) using the ethanol/formic acid extraction protocol according to the manufacturer’s instructions and partial *rpoB* sequencing and phylogenetic comparison to reference strains as described by Nicholson *et al*. [3] in BioNumerics v 6.6 (Applied Maths, Austin, TX, USA). Antimicrobial susceptibility testing of amikacin, ceftazidime, ciprofloxacin, gentamicin, levofloxacin, meropenem, piperacillin/tazobactam, tetracycline, tobramycin and trimethoprim/sulfamethoxazole was performed by the agar diffusion method according to CLSI guidelines (CLSI M07-A10) [24] on all isolates. The minimum inhibitory concentrations (MICs) were interpreted based on CLSI breakpoints for other non-* Enterobacteriaceae* (CLSI M100-S25) [25]. Although typically reserved for Gram-positive bacteria, vancomycin has been used as a treatment for *Elizabethkingia* bacteraemia [26, 27]. Therefore, susceptibility to vancomycin was determined by Etest (bioMérieux, Inc. Canada, St-Laurent, QC, Canada).

## Results and Discussion

The 2015–2016 Wisconsin outbreak of *E. anophelis* was unprecedented in its scale, with 66 laboratory-confirmed cases resulting in 19 deaths over 3 US states. According to WGS analysis, the outbreak isolates were genetically similar, suggesting a single source for these predominantly community-acquired cases that was never identified. *E. anophelis* had not previously been identified at our institution by conventional biochemical assays and/or MALDI-ToF MS. In light of the aforementioned outbreak, the identification of five isolates of *E. anophelis* from clinical specimens by PHOL was concerning. Given that these isolates were submitted from four different healthcare centres in the Greater Toronto Area (GTA), there was concern that these isolates could possibly represent an extension of the outbreak in Wisconsin, geographically located only approximately 690 kilometres away, with one outbreak case in Michigan, which borders Ontario. Alternatively, the isolates could possibly signify a separate outbreak, or not be related.

**Fig. 1.**
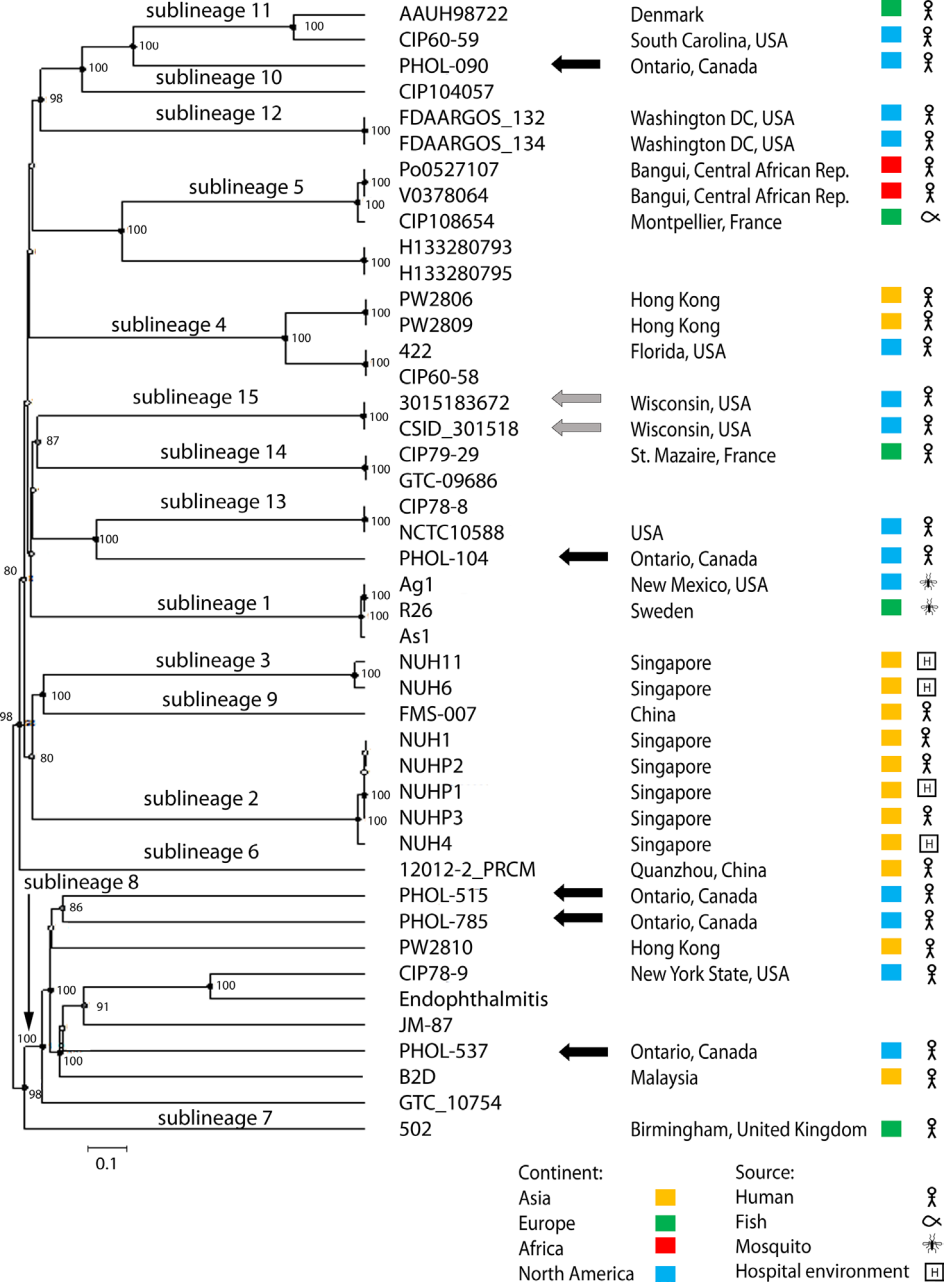
Genomic comparison using the UPGMA algorithm based on cgMLST profiles (1546 genes) of *E. anophelis* strains. The position of the 5 Ontario isolates (black arrows) is shown among a collection of 2 Wisconsin outbreak (grey arrows) isolates and 37 other strains representing the known diversity of the sublineages [6]. The scale bar corresponds to 10 % allelic mismatches (out of 1546 gene loci). Nodes with >80 % bootstrap support (*n*=1000 replicates) are indicated.

WGS analysis was instrumental in confirming and characterizing the 2015–2016 Wisconsin *E. anophelis* outbreak [4, 6]. Therefore, we sought to use WGS analysis to determine the clonal diversity of isolates to query the existence of a single-strain *E. anophelis* outbreak in Ontario. Genomic comparison analysis of cgMLST demonstrated that the Ontario isolates were not genetically similar to each other, and nor were they similar to the Wisconsin outbreak isolates. Two of the 5 Ontario isolates represented distinct sublineages, not previously encountered among a set of 39 representatives of formerly described sublineages of *E. anophelis* from several world regions. The remaining three were genetically dissimilar members of sublineage 8 (Fig. 1). Sublineages with multiple isolates are strongly supported, but their relative deep branching order is not. Therefore, we conclusively ruled out the presence of a single-strain *E. anophelis* outbreak in Ontario.

With the exception of the single isolate that was resistant to piperacillin/tazobactam, the antimicrobial susceptibility profiles of the five Ontario *E. anophelis* isolates were similar to each other and to those of the Wisconsin outbreak isolates [6]. Resistance to penicillins (except piperacillin/tazobactam), cephems, carbapenems, aminoglycosides and tetracyclines was observed, while the isolates remained susceptible to fluoroquinolones, especially levofloxacin, and the folate pathway inhibitor trimethoprim/sulfamethoxazole (Table 1). Interrogation of the genomes revealed multiple antimicrobial resistance genes (Table 1) as previously reported [4, 6, 9, 28]. These included two class A *β*-lactamases, three class B (metallo-) beta-lactamases, an aminoglycoside 6-nucleotidyltransferase, a chloramphenicol acetyltransferase and multiple efflux systems, all of which were present in each of the five Ontario isolates (Table 1). We identified the DNA gyrase and topoisomerase IV genes *gyrA*, *gyrB*, *parC* and *parE*, the target genes for fluoroquinolones in which mutations are often associated with resistance. Although the protein variants of these genes conferring fluoroquinolone resistance are likely not fully characterized, all five Ontario isolates contained a serine at position 83 of *gyrA*, which is associated with susceptibility, consistent with the phenotype of the isolates (Table 1) [6, 28]. Presumably, the protein variants of dihydrofolate reductase and dihydropteroate synthase found among the Ontario isolates confer susceptibility to trimethoprim/sulfamethoxazole, in accordance with their MICs (Table 1). As vancomycin has been used to treat *Elizabethkingia* infections [26, 27], we performed *in vitro* vancomycin MIC testing of the five Ontario *E. anophelis* isolates. The MICs ranged from 6 to 8 µg ml^−1^, but the clinical significance of this remains unclear since interpretative breakpoints for *Elizabethkingia* spp. to vancomycin do not exist.

**Table 1.** Summary of minimum inhibitory concentrations (MICs), susceptibilities to various antibiotics (S, susceptible; I, intermediate; R, resistant)^*a*^ ** and associated antibiotic resistance genes identified through WGS analysis of five Ontario *E. anophelis* clinical isolates

*a*, The interpretive criteria applied were those of CLSI for non-* Enterobacteriaceae* (CLSI M100-S25) [25].

*b*, Accession numbers of PHOL-104.

*c*, Protein homologues of resistance genes were identified using the hmmscan function of HMMER3 (http://hmmer.org/) against the Core ResFams database [18], the specialty genes for antibiotic resistance identified by PATRIC [19] following RAST annotation [20], and/or the Resistance Gene Identifier (Perfect and Strict hits only) of CARD [21, 22].

*d*, Protein variant model antibiotic resistance gene targets were identified following gene annotation by Prokka [16].

*e*, All isolates had a serine at position 83 conferring susceptibility to fluoroquinolones [6, 28].

Importantly, since *E. anophelis* had not been previously identified at PHOL, we questioned whether this signalled a potential increased incidence in *E. anophelis* infections or was a manifestation of improved bacterial identification techniques and improved sequence databases. Therefore, we performed a retrospective reidentification of an additional 17 isolates of *Elizabethkingia* received from 2010 to 2018; during this timeframe several identification methods were used. According to partial *rpoB* sequence analysis, which is considered to be preferable to 16S rDNA sequence analysis for discrimination to the species level for these organisms [3], clinical isolates of *E. anophelis* were in fact received by PHOL as early as 2010 (Table 2) but had not been identified correctly. In addition to 12 *E. anophelis *, we retrospectively identified clinical isolates of *E. meningoseptica* (*n*=2), *E. miricola* (*n*=2), ‘* E. bruuniana *’ (*n*=5) and ‘* E. ursingii *’ (*n*=1) (Table 2), which had previously been identified as *E. meningoseptica *, *E. miricola* or *Elizabethkingia* sp. As previously described [3, 11], Bruker MALDI-ToF MS in conjunction with their MALDI Biotyper Reference Library v7 was unable to differentiate *Elizabethkingia* spp. (Table 2), since scores >2.0 to multiple different species of *Elizabethkingia* were frequently obtained. Expansion of the spectrum database to include spectra from each species may allow for the differentiation of *E. anophelis* and *E. meningoseptica* in the future, but probably not *E. miricola *, ‘* E. bruuniana *’, ‘* E. ursingii *’ and ‘* E. occulta *’ [3]. Due to large phenotypic variability among *Elizabethkingia* strains of the same genomospecies, phenotypic testing is not recommended for species differentiation [3]. Although 16S rDNA sequencing was employed in our laboratory as a routine method for the identification of clinical bacterial isolates with ambiguous MALDI-ToF MS identifications during the timeframe of this study, we recognize retrospectively that it is not optimal for the species identification of *Elizabethkingia* due to sequence ambiguities deriving from multiple distinct 16S rDNA gene variants in a single genome and incongruences between 16S rDNA sequences and species determinations based on WGS data [3]. Retrospective reidentification of PHOL isolates suggested that the incidence of *E. anophelis* infections did not increase from 2010 to 2018, but rather that the bacterial identification techniques and algorithms improved, allowing for more accurate species determinations. Additionally, consistent with other reports [6, 11], most *Elizabethkingia* isolates demonstrated *in vitro* susceptibility to levofloxacin, piperacillin/tazobactam and trimethoprim/sulfamethoxazole, but were resistant to amikacin, tobramycin, ceftazidime, meropenem and tetracycline (Table 3).

**Table 2.** Isolates of *Elizabethkingia* (*n*=22) received by PHOL from 2010 to 2018 identified by biochemical assay/FAME analysis or 16S rDNA Sanger sequence analysis (original ID), Bruker MALDI-ToF MS and partial *rpoB* Sanger sequence analysis

Year	Original ID	MALDI-ToF MS ID^a^	*rpoB* ID^*b*^
2010	* E. meningoseptica* ^c^	* E. meningoseptica / E. miricola *	* E. anophelis *
2010	*E. meningoseptica^c^*	* E. meningoseptica / E. miricola *	* E. anophelis *
2011	*E. meningoseptica^c^*	* E. meningoseptica *	* E. anophelis *
2011	*E. meningoseptica^c^*	* E. meningoseptica / E. miricola *	* E. anophelis *
2011	*E. meningoseptica^c^*	* E. meningoseptica / E. miricola *	* E. anophelis *
2013	*E. meningoseptica^c^*	* E. meningoseptica / E. miricola *	* E. anophelis *
2013	*E. meningoseptica^c^*	* E. meningoseptica / E. miricola *	* E. anophelis *
2013	*E. meningoseptica^c^*	* E. meningoseptica *	* E. meningoseptica *
2014	*E. meningoseptica^c^*	* E. miricola *	*‘ E. bruuniana ’*
2015	* Elizabethkingia sp*.^*c*^	* E. miricola *	* E. miricola *
2015	*E. meningoseptica^c^*	* E. meningoseptica *	* E. meningoseptica *
2015	* E. miricola /meningoseptica^d^*	* E. miricola / E. meningoseptica *	*‘ E. bruuniana ’*
2016	* E. miricola /meningoseptica^d^*	* E. miricola *	*‘ E. bruuniana ’*
2016	*E. anophelis^d^*	* E. meningoseptica / E. miricola *	* E. anophelis *
2017	*E. miricola^d^*	* E. miricola *	*‘ E. bruuniana ’*
2017	* Elizabethkingia* sp.^*d*^	* E. meningoseptica / E. miricola *	* E. anophelis *
2017	*E. anophelis^d^*	* E. meningoseptica / E. miricola *	* E. anophelis *
2017	* E. miricola /meningoseptica^d^*	* E. miricola *	* E. miricola *
2018	*E. anophelis^d^*	* E. meningoseptica *	* E. anophelis *
2018	* Elizabethkingia* sp.^*d*^	* E. meningoseptica / E. miricola *	* E. anophelis *
2018	*E. miricola^d^*	* E. miricola *	*‘ E. bruuniana ’*
2018	* Elizabethkingia* sp.^*d*^	* E. miricola / E. meningoseptica *	*‘ E. ursingii ’*

*a*, The MALDI-ToF MS ID was generated by comparison of Bruker MALDI-ToF mass spectra obtained using the ethanol/formic acid extraction protocol against the MALDI Biotyper Reference Library v7 (Bruker Daltonics).

*b*, The *rpoB* ID was performed by partial *rpoB* Sanger sequencing and phylogenetic comparison to reference strains as described by Nicholson *et al*. [3].

*c*, Original identification was performed using biochemical assays and FAME analysis.

*d*, Original identification was performed by Bruker MALDI-TOF MS and partial 16S rDNA sequence analysis; 16S rDNA sequence analysis was considered to be the ‘gold standard’ method.

**Table 3.** Percentage of *Elizabethkingia* isolates non-susceptible to various antibiotics*

Antibiotic	* E. anophelis* (*n*=12)	* E. meningoseptica* (*n*=2)	‘* E. bruuniana *’ (*n*=5)	* E. miricola* (*n*=2)	‘* E. ursingii *’ (*n*=1)
Amikacin	100	100	100	100	100
Ceftazidime	100	100	100	100	100
Ciprofloxacin	41.7	50	40	50	0
Gentamicin	100	100	100	100	0
Levofloxacin	8.3	50	0	50	0
Meropenem	100	100	100	100	100
Piperacillin/tazobactam†	8.3	0	0	0	100
Tetracycline	100	100	100	100	100
Tobramycin	100	100	100	100	100
Trimethoprim/sulfamethoxazole‡	0	0	0	0	0

*The interpretive criteria applied were those of CLSI for non-* Enterobacteriaceae* (CLSI M100-S25) [25].

†The concentration of tazobactam was 4 µg ml^−1^ constant.

‡The ratio of trimethoprim to sulfamethoxazole was 1 to 19.

In conclusion, we used genome sequencing to demonstrate that a multiclonal population of *E. anophelis* caused infections in Ontario patients, thus conclusively refuting the existence of a single-strain outbreak. Because of its powerful predictive value, WGS will undoubtedly be used with increasing frequency to rapidly investigate case clusters to either confirm or rule out single-strain outbreaks, as well as for genomic characterization of other emerging trends in clinical microbiology laboratory science. Additionally, the identification of *Elizabethkingia* to the species level remains a challenge, even with improved technologies. In lieu of WGS analysis, targeted *rpoB* sequence analysis may represent the best option for discriminating *Elizabethkingia* to the species level [3].

## References

[1] Bruun B, Bernardet J, Whitman WB (2015). Elizabethkingia. Bergey's Manual of Systematics of Archaea and Bacteria.

[2] King EO (1959). Studies on a group of previously unclassified bacteria associated with meningitis in infants. Am J Clin Pathol.

[3] Nicholson AC, Gulvik CA, Whitney AM, Humrighouse BW, Graziano J (2018). Revisiting the taxonomy of the genus *Elizabethkingia* using whole-genome sequencing, optical mapping, and MALDI-TOF, along with proposal of three novel *Elizabethkingia* species: *Elizabethkingia* bruuniana sp. nov., *Elizabethkingia* ursingii sp. nov., and *Elizabethkingia* occulta sp. nov. Antonie Van Leeuwenhoek.

[4] Breurec S, Criscuolo A, Diancourt L, Rendueles O, Vandenbogaert M (2016). Genomic epidemiology and global diversity of the emerging bacterial pathogen *Elizabethkingia anophelis*. Sci Rep.

[5] Jean SS, Lee WS, Chen FL, Ou TY, Hsueh PR (2014). *Elizabethkingia meningoseptica*: an important emerging pathogen causing healthcare-associated infections. J Hosp Infect.

[6] Perrin A, Larsonneur E, Nicholson AC, Edwards DJ, Gundlach KM (2017). Evolutionary dynamics and genomic features of the *Elizabethkingia anophelis* 2015 to 2016 Wisconsin outbreak strain. Nat Commun.

[7] Balm MN, Salmon S, Jureen R, Teo C, Mahdi R (2013). Bad design, bad practices, bad bugs: frustrations in controlling an outbreak of *Elizabethkingia meningoseptica* in intensive care units. J Hosp Infect.

[8] Moore LS, Owens DS, Jepson A, Turton JF, Ashworth S (2016). Waterborne *Elizabethkingia meningoseptica* in Adult Critical Care. Emerg Infect Dis.

[9] Teo J, Tan SY, Liu Y, Tay M, Ding Y (2014). Comparative genomic analysis of malaria mosquito vector-associated novel pathogen *Elizabethkingia anophelis*. Genome Biol Evol.

[10] Lau SK, Chow WN, Foo CH, Curreem SO, Lo GC (2016). *Elizabethkingia anophelis* bacteremia is associated with clinically significant infections and high mortality. Sci Rep.

[11] Han MS, Kim H, Lee Y, Kim M, Ku NS (2017). Relative Prevalence and Antimicrobial Susceptibility of Clinical Isolates of *Elizabethkingia* Species Based on 16S rRNA Gene Sequencing. J Clin Microbiol.

[12] Altschul SF, Gish W, Miller W, Myers EW, Lipman DJ (1990). Basic local alignment search tool. J Mol Biol.

[13] Benson DA, Cavanaugh M, Clark K, Karsch-Mizrachi I, Ostell J (2018). GenBank. Nucleic Acids Res.

[14] CLSI (2018). Interpretive Criteria for Identification of Bacteria and fungi by DNA target sequencing.

[15] Bankevich A, Nurk S, Antipov D, Gurevich AA, Dvorkin M (2012). SPAdes: a new genome assembly algorithm and its applications to single-cell sequencing. J Comput Biol.

[16] Seemann T (2014). Prokka: rapid prokaryotic genome annotation. Bioinformatics.

[17] HMMER (2015). HMMER: biosequence analysis using profile hidden Markov models v3.1b2.

[18] Gibson MK, Forsberg KJ, Dantas G (2015). Improved annotation of antibiotic resistance determinants reveals microbial resistomes cluster by ecology. ISME J.

[19] Wattam AR, Abraham D, Dalay O, Disz TL, Driscoll T (2014). PATRIC, the bacterial bioinformatics database and analysis resource. Nucleic Acids Res.

[20] Aziz RK, Bartels D, Best AA, DeJongh M, Disz T (2008). The RAST server: rapid annotations using subsystems technology. BMC Genomics.

[21] Jia B, Raphenya AR, Alcock B, Waglechner N, Guo P (2017). Card 2017: expansion and model-centric curation of the comprehensive antibiotic resistance database. Nucleic Acids Res.

[22] McArthur AG, Waglechner N, Nizam F, Yan A, Azad MA (2013). The comprehensive antibiotic resistance database. Antimicrob Agents Chemother.

[23] Weyant RS, Wayne C, Weaver RE, Hollis DG, Jordan JG (1996). Identification of Unusual Pathogenic gram-negative aerobic and facultatively anaerobic bacteria.

[24] CLSI (2015). Methods for dilution antimicrobial susceptibility tests for bacteria that grow aerobically.

[25] CLSI (2015). Performance standards for antimicrobial susceptibility testing.

[26] Jean SS, Hsieh TC, Ning YZ, Hsueh PR (2017). Role of vancomycin in the treatment of bacteraemia and meningitis caused by *Elizabethkingia meningoseptica*. Int J Antimicrob Agents.

[27] Figueroa Castro CE, Johnson C, Williams M, VanDerSlik A, Graham MB (2017). *Elizabethkingia anophelis*: clinical experience of an academic health system in Southeastern Wisconsin. Open Forum Infect Dis.

[28] Lin JN, Lai CH, Yang CH, Huang YH, Lin HH (2017). Genomic features, phylogenetic relationships, and comparative genomics of *Elizabethkingia anophelis* strain EM361-97 isolated in Taiwan. Sci Rep.

